# Long-Term Assessment of Periodontal Tissues after Corticotomy-Assisted Orthodontic Arch Expansion

**DOI:** 10.3390/jcm10235588

**Published:** 2021-11-27

**Authors:** Magdalena Ewa Sulewska, Amelia Baczewska, Beata Bugała-Musiatowicz, Emilia Waszkiewicz-Sewastianik, Jan Krzysztof Pietruski, Małgorzata Pietruska

**Affiliations:** 1 Department of Periodontal and Oral Mucosa Diseases, Medical University of Bialystok, ul. Waszyngtona 13, 15-269 Białystok, Poland; amelia.baczewska@umb.edu.pl (A.B.); malgorzata.pietruska@umb.edu.pl (M.P.); 2 Dental Practice, ul. Żeromskiego 1A/1U, 15-349 Białystok, Poland; beatryczeort@o2.pl; 3 Dental Practice, ul. Waszyngtona 1/34, 15-269 Białystok, Poland; milkanet@interia.pl (E.W.-S.); janpietruski@wp.pl (J.K.P.)

**Keywords:** orthodontic arch expansion, corticotomy, soft tissues

## Abstract

Objectives: The aim of the study was the long-term assessment of the condition of periodontal tissues after corticotomy-assisted orthodontic expansion in patients with transverse maxillary deficiency. Materials and Methods: The study included a group of 18 adults (9 women, 9 men) aged between 24 and 40 years who were at least 5 years post treatment. The following parameters were assessed: the full mouth plaque index (FMPI), full mouth bleeding on probing (FMBOP), probing depth (PD), clinical attachment level (CAL), gingival recession height (GR), recession width (RW), papilla height (PH), papilla width (PW), bone sounding (BS), phenotype, and KT. Results: During examination performed at least 5 years after the completion of orthodontic treatment, the values of PD and CAL were found to be considerably decreased compared to the examination one year post treatment (PD: −0.23; 95% Cl: −0.29, −0.16) (CAL: −0.04; 95% Cl: −0.17, 0.10). The other parameters—FMPI, FMBOP, GR, RW, PH, PW, BS, phenotype, and KT—did not change significantly. Conclusions: Corticotomy-assisted orthodontic arch expansion does not have a negative effect on the periodontium in long-term observations. Clinical Relevance: Orthodontic arch expansion can lead to bone dehiscence and gingival recession. Long-term observations revealed that corticotomy-assisted orthodontic expansion of the upper arch is not followed by negative changes in periodontal status.

## 1. Introduction

Maintaining a healthy periodontium is the key to maintaining a fully functional stomatognathic system. All procedures should be carried out in a way that prevents damage to the structures of periodontal tissues and enables the beneficial effects after the applied treatment to be maintained in the long term. Orthodontic treatment of patients with transverse maxillary deficiency can lead to adverse changes in the bone and soft tissues. As a result of the expansion of the dental arch, particularly in patients with the thin phenotype, it is highly probable to develop bone dehiscence and gingival recession [1,2,3,4,5]. The consequences of gingival recession, in addition to the aesthetic problem, may include loss of hard dental tissues and tooth sensitivity [6,7]. The treatment of this type of complication requires significant financial resources and additionally burdens the patient. Furthermore, it does not offer the possibility of reconstructing the lost bone or soft tissues, which may generate further adverse changes in the long-term observations [8]. Therefore, in order to limit the negative effects of arch expansion on periodontal tissues, additional methods supporting orthodontic treatment are sought. A basic type of treatment, especially recommended to patients with a thin phenotype, is gingival augmentation with the use of autogenous bone grafts or biomaterials as a form of prevention of gingival recession [9,10]. A completely different concept is corticotomy, which consists of the cutting of buccal and/or palatal compact bone plate, generating the regional acceleratory phenomenon (RAP). RAP is a cascade of physiological events leading to increased bone turnover accompanied by demineralization and the formation of a new bone at the site of injury [11,12,13,14]. Due to this phenomenon, when orthodontic force is applied, not only the tooth but also the adjacent demineralized bone matrix is displaced, which potentially reduces the risk of bone dehiscence and the subsequent gingival recession [15,16,17,18,19,20,21,22]. It should be remembered that bone apposition is also stimulated by properly performed orthodontic treatment [23].

The currently available literature data do not provide a clear answer as to whether the use of corticotomy prior to orthodontic arch expansion prevents adverse bone and soft tissue changes [24,25,26,27,28,29,30,31,32,33,34,35]. Moreover, there are no long-term data evaluating periodontal changes after this type of treatment.

In the light of the above, the aim of this study was to assess the periodontium condition at least five years after the combined surgical and orthodontic treatment of patients with maxillary narrowing.

## 2. Materials and Methods

The study was designed as a case series study. The study involved 20 patients treated in the years 2011–2014. Patients with transverse maxillary deficiency, scheduled for corticotomy-supported orthodontic treatment of the upper dental arch, qualified for the study. The sample size calculation was made in G*POWER software using 0.2 effect size and power = 0.9. The aim of performing corticotomy was to prevent bone dehiscence and gingival recession.

The inclusion criteria were as follows: voluntary participation, adult (>18 years old), non-smoker, generally healthy, with malocclusion with transverse maxillary deficiency with indications for upper arch expansion, good oral hygiene and motivation at screening quantified as: full mouth plaque index (FMPI) <20%, full mouth bleeding on probing (FMBOP) <20%. The exclusion criteria were the following diseases or conditions: periodontal disease and oral mucosa lesions, bisphosphonate and long-term corticosteroid therapy, current therapy with anti-epileptic drugs, contraceptives, estrogen, antihistamine drugs, calcitonin, vitamin D, alcohol and/or drug addiction, the presence of periapical endo-periolesions, severe gingival recession, pregnancy, breast feeding, previous orthodontic treatment and root resorption. Within the study group, the following malocclusions were diagnosed: Class I with crowding—six patients; Class I with lateral crossbite—three patients; Class I with bilateral crossbite—two patients; Class II Division 1—one patient; Class II Division 1 with anterior open bite—two patients; Class II Division 2—one patient; and Class III with a skeletal relationship—five patients.

The surgery was done in maxilla under local anesthesia with 4% articaine (Ubistesin forte, 3 M ESPE, Maplewood, MN, USA). The mucoperiosteal flap was elevated up to the point above the apical parts of roots following modified papilla preservation technique as well as performing vertical releasing incisions [36]. Then, osteotomy of the buccal cortical plate of the alveolar process was performed by using OTS7, OTS7–4, and OTS7-3 ultrasound tips of the piezosurgery device (Mectron S.p.A., Carasco, Italy). The extension of the osteotomy was determined by the mesio-distal dimension of the teeth roots as well as by the position of the apexes of roots. In order to avoid interproximal bone pick resorption, the vertical cuts ended 5 mm apically from the crest and then spread in a Y-shape towards the neighboring teeth. The horizontal corticotomy was performed approximately 2–4 mm apically above the root apexes. The depth of the cuts was limited to the thickness of the cortical plate. The repositioned flap was sutured with non-resorbable monofilament 5.0 and 6.0 sutures (Resolon, Resorba Medical GmbH, Nümberg, Germany). One gram of amoxicillin 2×/day for 7 days, 200 mg of ibuprofen 3×/day, and mouth rinsing with chlorhexidine (0.10% Eludril, Pierre Fabre Sante, Paris, France) 2×/day were prescribed, and gentle tooth brushing using an ultra-soft post-surgical toothbrush and the roll technique in the surgical area for two weeks after the surgery was recommended to the patients. The supragingival plaque was cleaned out 7 and 14 days after the surgery. The sutures were removed 14 days post-op, and tooth brushing with a soft toothbrush and the roll technique was recommended. The detailed methodology and post-treatment results are presented in the publication from 2018 [25].

A check-up was performed 5–8 years after corticotomy (on average, after 6.5 years). The examination was participated in by 18 subjects (9 women, 9 men) aged from 26 to 46 years. Two patients (1 female and 1 male) did not attend the examination due to the COVID-19 pandemic. All clinical examinations were performed by the same doctor.

The following parameters were assessed: the full mouth plaque index (FMPI), full mouth bleeding on probing (FMBOP), probing depth (PD), clinical attachment level (CAL), gingival recession height (GR), recession width (RW), papilla height (PH), papilla width (PW), bone sounding (BS), phenotype and keratinized tissue (KT).

The measurements were performed using a manual PCP UNC 15 periodontal probe (Hu-Friedy, Chicago, IL, USA) by one calibrated investigator. All measurements were rounded to the nearest 0.5 mm.

Phenotype—gingival thickness was measured under anaesthesia at mid-facial aspect of the tooth on a long axis 1 mm apically from the bottom of the sulcus with the use of K-file 25 ISO with a silicone marker [37]. Keratinized tissue (KT) was measured from the most apical point of gingival margin to the mucogingival junction.

The study was conducted in accordance with the Helsinki Declaration of 1975, as revised in 2000, and was reviewed and approved by the local ethical committee (Ethics Committee No.: R-I-002/344/2011).

## 3. Statistical Analysis

The variables were described by the parameters of descriptive statistics, i.e., arithmetic mean, standard deviation, median, lower and upper quartile, and minimum and maximum value. The normality of distribution was tested using the Shapiro–Wilk test. ANOVA, MANOVA with Tukey’s post hoc HSD test, or Friedman’s test with Dunn’s multiple comparisons test with Bonferroni correction (depending on the assumptions fulfilled) were used for comparing the three groups. For differences between the measurements (one year vs. a minimum of 5 years after the treatment), the mean values with 95% confidence interval and standard deviation are provided. For statistical analyses, a significance level of *p* < 0.05 was assumed. The analyses were performed using the PQStat program, version 1.8.0.476.

## 4. Results

During the follow-up examination, 142 teeth were examined—one molar had been previously extracted due to endodontic complications.

It was found that one year after corticotomy and at least five years later, FMPI and FMBOP values were comparable (Table 1).

In the examinations performed at least five years post treatment, we observed a significant reduction in PD and CAL compared to the examination conducted one year after treatment. The values of PD decreased from 2.48 ± 0.51 to 2.28 ± 0.48 mm, and those of CAL decreased from 2.49 ± 0.51 to 2.48 ± 0.86 mm (Table 2).

**Figure 1:**
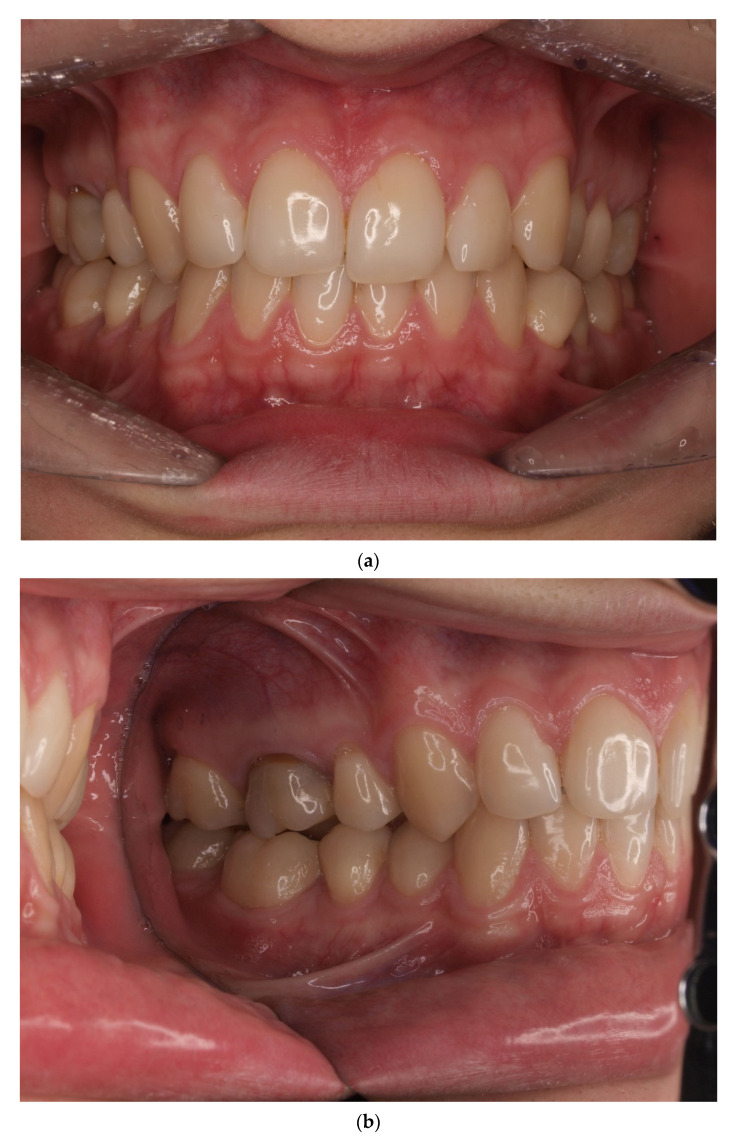

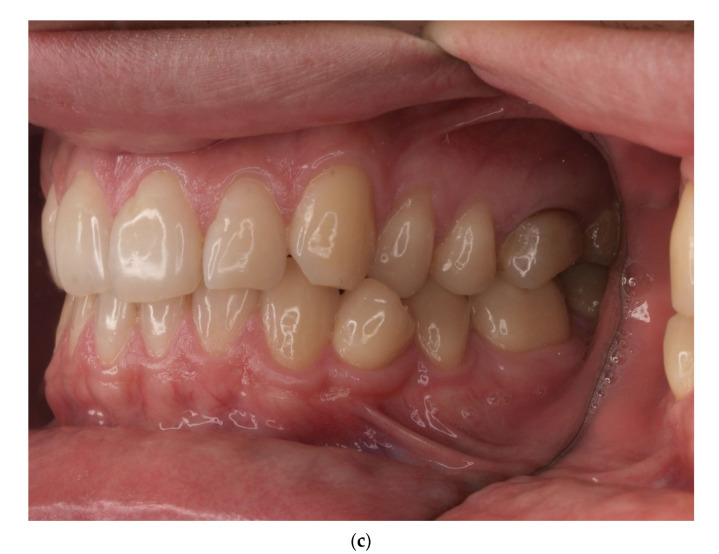
(**a**) Status before orthodontic treatment—lateral crossbite (left side). (**b**) Status before orthodontic treatment—right side. (**c**). Status before orthodontic treatment—left side.

**Figure 2:**
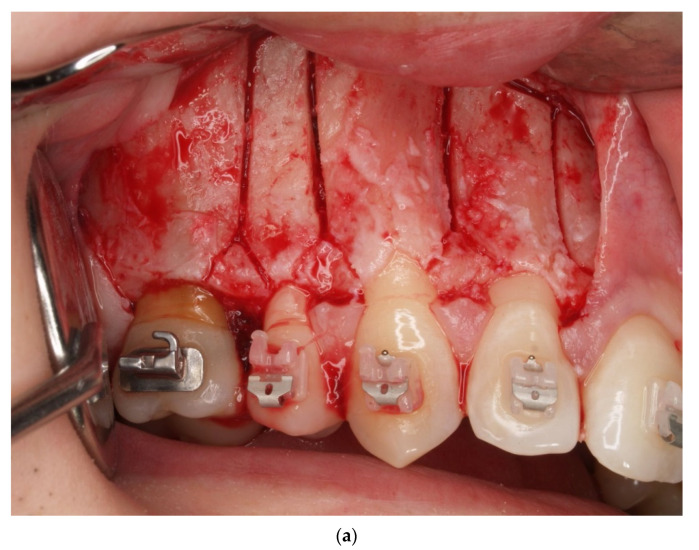

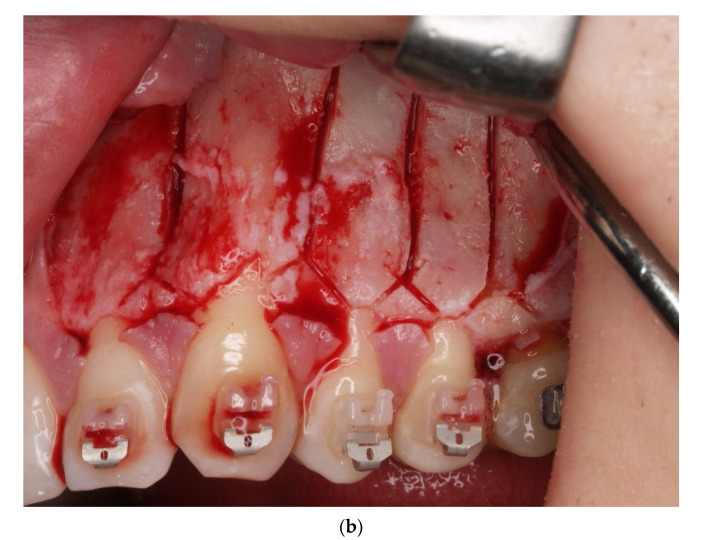
(**a**) Corticotomy in the area of the upper lateral incisor, canine and premolars. Incision of the cortical plate in the interdental spaces and above the apexes of the teeth—right side. (**b**) Corticotomy in the area of the upper canine and premolars. Incision of the cortical plate in the interdental spaces and above the apexes of the teeth—left side.

**Figure 3:**
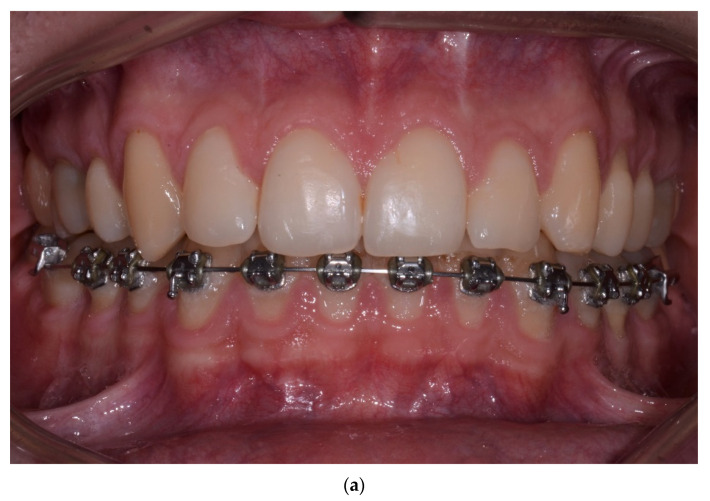

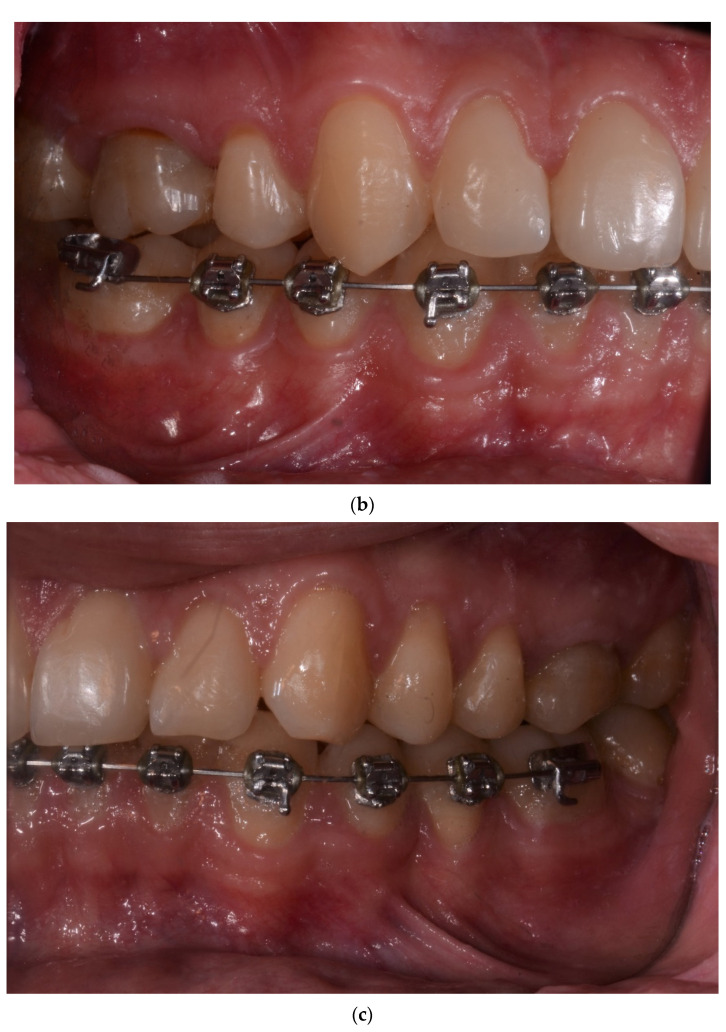
(**a**) Status one year after orthodontic treatment completion. There are no evident adverse changes in the position of the gingival margin after labial tooth movement. (**b**) Status one year after orthodontic treatment completion. There are no evident adverse changes in the position of the gingival margin after labial tooth movement—right side. (**c**) Status one year after orthodontic treatment completion. There are no evident adverse changes in the position of the gingival margin after labial tooth movement—left side.

**Figure 4:**
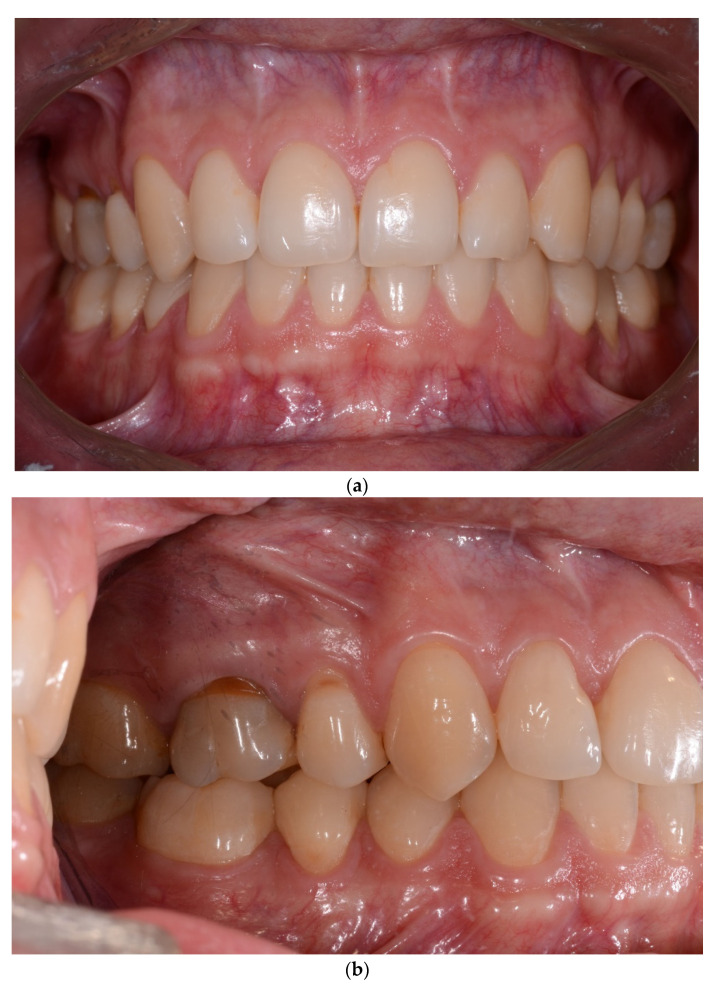

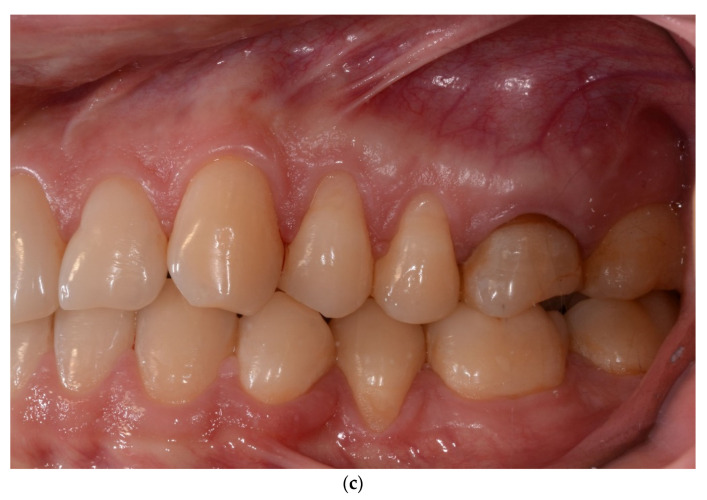
(**a**) Status six years after orthodontic treatment completion. The position of the gingival margin one year and six years after treatment is comparable. (**b**) Status six years after orthodontic treatment completion. The position of the gingival margin one year and six years after treatment is comparable—right side. (**c**) Status six years after orthodontic treatment completion. The position of the gingival margin one year and six years after treatment is comparable—left side.

One year post treatment, 8/159 (5.03%) gingival recessions of Miller Class I were found. In the follow-up examination, the number of gingival recessions increased to 9/142 (6.34%). The values of all the other analyzed parameters, viz: GR, RW, PH, PW, BS, phenotype and KT, did not change significantly during the evaluated observation period. Detailed numerical data related to these parameters are included in Table 3. Figure 1, Figure 2, Figure 3 and Figure 4 present the clinical status of one of the patients—before the treatment, during corticotomy procedures, as well as one year and six years after the corticotomy procedure.

## 5. Discussion

The aim of the presented study was to analyze possible changes in the periodontium after corticotomy-assisted orthodontic expansion of the maxillary dental arch in a long-term observation. The clinical examination involved evaluation of numerous parameters which enabled a detailed qualitative and quantitative assessment of the soft tissues as well as the position of the margin of the buccal cortical plate. No adverse changes were found in the periodontal tissues—the values of most parameters remained similar to those obtained one year after the procedure. Moreover, the values of PD and CAL were significantly reduced, by 0.2 and 0.01 mm, respectively, which emphasizes the stability of periodontal tissues over time.

The available literature lacks detailed multidirectional studies evaluating periodontal changes after combined surgical and orthodontic treatment. Nevertheless, most publications report the lack of negative effects of this type of treatment on the periodontium, based on such parameters as PD, CAL, BOP, or the number of gingival recessions [22,30,31,33,38,39,40,41,42,43,44,45,46]. Data published in a systematic review in 2020 indicate that PD values in short-term observations not exceeding 20 months decrease by 0.32–0.58 mm [47]. According to some authors, the reduction in this parameter is statistically significant at 0.58, 0.427 and 0.36 mm [27,28,48]. Other researchers reported an insignificant reduction in PD by 0.2, 0.323 or 0.25 mm [24,31,35].

The literature data concerning CAL changes after the discussed therapeutic procedure are very limited. The available data suggest that either no changes in CAL values were observed after treatment, or the reduction in CAL in short-term observations was 0.1 mm [24,32,33].

Our study revealed that the only adverse change over time was the appearance of 1 gingival recession of 2 mm in height. The recession appeared on the tooth adjacent to the extracted one; therefore, it can be assumed that it was the result of soft tissue deformation associated with tooth extraction. In contrast, the increase in mean height of the gingival recession was 0.12 mm and was not statistically significant. The obtained results may be related to the use of appropriate minimally invasive techniques aimed at preserving gingival papillae intact [49]. The absence of newly formed gingival recessions in short-term observations of three and four months was also reported by other authors [30,32,33]. Studies comparing the orthodontic-surgical procedure with classical orthodontic treatment are also noteworthy. It has been demonstrated that after orthodontic treatment with corticotomy, the value of GR decreased insignificantly by 0.07 mm (from 0.49 ± 0.26 mm to 0.42 ± 0.3 mm), while classical orthodontic treatment entailed its insignificant increase by 0.64 mm (from 0.55 ± 0.31 to 1.19 ± 0.24 mm) [31].

It should be emphasized that the discussed therapeutic procedure had no negative effects on the quantity and quality of soft tissues, i.e., their phenotype (gingival thickness) and KT, in the long-term observations. These parameters improved significantly a year after the procedure compared to their baseline value (phenotype by 0.32 mm, KT by 0.1 mm) and remained at a comparable level for an average of 6.5 years. These results seem optimistic compared to those obtained after classical orthodontic treatment. Indeed, a significant reduction in gingival thickness was demonstrated in the region of the maxillary (by 0.15–0.35 mm) and mandibular (by 0.18–0.3 mm) anterior teeth after their displacement in the sagittal plane [50]. A mean reduction in the KT height of 0.25 mm immediately after conventional treatment has also been reported [51]. According to other authors, a significant reduction in KT width occurred only in maxillary lateral incisors after protrusive movement, giving rise to the hypothesis that there is a positive relationship between sagittal tooth displacement and KT width [50].

A mean reduction in the KT height of 0.25 mm has also been reported immediately after conventional treatment [51]. According to other authors, a significant decrease in KT width occurred only with maxillary lateral incisors after protrusive movement, leading to the conclusion that there is a positive relationship between sagittal tooth displacement and KT width [50]. This thesis was confirmed by Wang et al. [52], who reported that displacement of teeth during orthodontic treatment affects the width of the keratinized gingiva, and an increase in positive torque results in a greater likelihood of reducing the width of the keratinized gingiva. However, there are no literature data evaluating gingival thickness after combined surgical and orthodontic treatment.

The available literature also contains negative statements regarding orthodontic–surgical treatment. Some authors claim that adverse effects of this procedure include increased gingival recession and alveolar bone loss due to the existing periodontal diseases or premature removal of sutures [15,47,53]. These statements may seem rather controversial, especially in view of the fact that treatment is performed in the presence of coexisting inflammation. According to data obtained by other researchers as well as our own experience, such factors as appropriate patient selection, oral hygiene regime, absence of inflammation, and the precision of the surgical procedure minimize the risk of the above-mentioned problems. Therefore, corticotomy-assisted orthodontic arch expansion appears to be a safe procedure that ensures long-term stability of periodontal tissues. Several authors even suggest the possibility of an osteogenic effect of alveolar corticotomy [54,55,56,57].

A major limitation of this study is the lack of a control group, the small sample size and the single examiner. The lack of a control group results from ethical constraints. The approval of the bioethics committee was obtained for the corticotomy procedure and not for other procedures protecting periodontal tissues, such as soft tissue augmentation. Thus, it is difficult to explain whether or not the change in PD and CAL parameters is due to the corticotomy procedure performed. Appropriate qualification of patients as well as appropriate microsurgical techniques contributed to the obtained results. It is difficult to obtain a large group of patients with the presented orthodontic defect and willing to start complex treatment. This is due to both economic constraints and the specificity of the performed treatment. The procedures applied by us are associated with a large number of visits and fear of surgery.

The favourable changes in the periodontium may result from the process of tissue remodelling, the expression of which is the highest immediately after the surgical procedure [11,33,40,58,59,60]. However, our results should be analyzed cautiously due to the fact that they constitute the first long-term data evaluating the condition of periodontium after combined surgical and orthodontic treatment. Nevertheless, these results highlight the fact that while making a treatment plan for patients with transverse maxillary deficiency, the corticotomy procedure should be considered as a potential form of preventing the development of bone dehiscence and gingival recession.

## Figures and Tables

**Table 1:** Full mouth plaque index (FMPI) and full mouth bleeding on probing (FMBOP) before, 1 year and a minimum of 5 years after orthodontic treatment.

Parameter	[%]	Time of Observation	Difference between 1 Year and a Minimum of 5 Years Post-op	*p*-Value	Mean Diff. (95% Cl) between 1 Year and a Minimum of 5 Years Post-op
FMPI	x ± SD	Baseline 17.33 ± 2.11 1 year 16.90 ± 2.25 5 years 17.20 ± 2.18	+0.30%	*p* = 0.699 *	0.42 (−0.91, 1.75)
FMBOP	x ± SD	Baseline 13.44 ± 1.87 1 year 13.24 ± 1.75 5 years 13.32 ± 1.46	+0.08%	*p* = 0.724 **	0.26 (−0.61, 1.12)

x ± SD—mean values and standard deviation; Mean diff.—mean difference; Cl—confidence interval; * Tukey’s post hoc HSD test (ANOVA); ** Tukey’s post hoc HSD test (MANOVA).

**Table 2:** Probing depth (PD), clinical attachment level (CAL), bone sounding (BS) before, 1 year and a minimum of 5 years after the corticotomy-assisted orthodontic treatment.

Parameter	[mm]	Time of Observation	Difference between 1 Year and a Minimum of 5 Years Post-op	*p*-Value	Mean Diff. (95% Cl) between 1 Year and a Minimum of 5 Years Post-op
PD	x ± SD	Baseline 2.74 ± 0.57 1 year 2.48 ± 0.51 5 years 2.28 ± 0.48	−0.20 mm	*p* < 0.0001 *	−0.23 (−0.29, −0.16)
CAL	x ± SD	Baseline 2.75 ± 0.57 1 year 2.49 ± 0.51 5 years 2.48 ± 0.86	−0.01 mm	*p* = 0.0350 *	−0.04 (−0.17, 0.10)
BS	x ± SD	Baseline 4.76 ± 0.94 1 year 4.49 ± 0.77 5 years 4.29 ± 0.70	−0.20 mm	*p* = 0.2430 *	−1.11 (−1.43, −0.79)

x ± SD—mean values and standard deviation; Mean diff.—mean difference; Cl—confidence interval; * Dunn’s multiple comparisons test with Bonferroni correction (Friedman).

**Table 3:** Biotype, papilla width (PW), papilla height (PH), gingival recession (GR), recession width (RW) and keratinized tissue (KT) before, 1 year and a minimum of 5 years after the corticotomy–assisted orthodontic treatment.

Parameter	[mm]	Time of Observation	Difference between 1 Year and a Minimum of 5 Years Post-op	*p*-Value	Mean Diff. (95% Cl) between 1 Year and 5 Years Post-op
Phenotype	x ± SD	Baseline 1.71 ± 0.52 1 year 2.03 ± 0.47 5 years 1.99 ± 0.50	−0.04 mm	*p* = 1 *	−0.02 (−0.05, 0.02)
PW	x ± SD	Baseline 3.75 ± 0.92 1 year 3.54 ± 1.50 5 years 4.03 ± 0.89	+0.49 mm	*p* = 0.103 *	0.38 (0.18, 0.58)
PH	x ± SD	Baseline 4.82 ± 1.16 1 year 4.00 ± 1.60 5 years 4.29 ± 3.56	+0.29 mm	*p* = 0.057 *	0.19 (−0.40, 0.78)
GR	x ± SD	Baseline 0.13 ± 0.47 1 year 0.07± 0.32 5 years 0.19 ± 0.79	+0.12 mm	*p* = 1 *	0.11 (0.01, 0.21)
RW	x ± SD	Baseline 0.21 ± 0.75 1 year 0.10 ± 0.49 5 years 0.14 ± 0.59	+0.04 mm	*p* = 1 *	0.02 (−0.07, 0.12)
KT	x ± SD	Baseline 5.02 ± 1.79 1year 5.12 ± 1.78 5 years 5.12 ± 1.72	0.00 mm	*p* = 1 *	−0.03 (−0.23, 0.18)

x ± SD—mean values and standard deviation; Mean diff.—mean difference; Cl—confidence interval; * Dunn’s multiple comparisons test with Bonferroni correction (Friedman).

## Data Availability

The data presented in this study are available in the article.
